# Compounds from Gum Ammoniacum with Acetylcholinesterase Inhibitory Activity

**DOI:** 10.3797/scipharm.1306-16

**Published:** 2013-08-12

**Authors:** Hamid-Reza Adhami, Johannes Lutz, Hanspeter Kählig, Martin Zehl, Liselotte Krenn

**Affiliations:** 1 Department of Pharmacognosy, University of Vienna, Althanstraße 14, 1090 Vienna, Austria.; 2 Institute of Organic Chemistry, University of Vienna, Währingerstraße 38, 1090 Vienna, Austria.

**Keywords:** *Dorema ammoniacum*, Acetylcholinesterase inhibition, Doremone A, Dshamirone, Ammoresinol, Spiro-sesquiterpenoidic chromane-2, 4-dione

## Abstract

The use of herbal medicinal preparations in dementia therapy has been studied based on experience from traditional medicine. A dichloromethane extract of gum ammoniacum, the gum-resin from *Dorema ammoniacum* D. Don had shown acetylcholinesterase (AChE) inhibitory activity in a previous study. The aim of this study was the isolation and characterization of the active compounds from this resin. The extract was investigated by a respective colorimetric microplate assay and the active zones were identified via TLC bioautography and isolated using several chromatographic techniques. The structures of the active components were characterized by one- and two-dimensional ^1^H and ^13^C NMR spectroscopy and mass spectrometry as (2′*S*,5′*S*)-2′-ethenyl-5′-(3-hy-droxy-6-methyl-4-oxohept-5-en-2-yl)-7-methoxy-2′-methyl-4*H*-spiro[chromene-3,1′-cyclopentane]-2,4-dione (**1**), which is an analogue of doremone A and a new natural compound, and as (2′*S*,5′*R*)-2′-ethenyl-5′-[(2*R*,4*R*)-4-hydroxy-6-methyl-3-oxohept-5-en-2-yl]-7-methoxy-2′-methyl-4*H*-spiro[chromene-3,1′-cyclo-pentane]-2,4-dione (**2** = doremone A), (4*E*,8*E*)-1-(2,4-dihydroxyphenyl)-5,9,13-trimethyltetradeca-4,8,12-trien-1-one (**3** = dshamirone), and 4,7-dihydroxy-3-[(2*E*,6*E*)-3,7,11-trimethyldodeca-2,6,10-trien-1-yl]-2*H*-chromen-2-one (**4** = am-moresinol). Dshamirone turned out to be the most active compound with an IC_50_ value for AChE inhibitory activity of 23.5 μM, whereas the other substances showed weak activity. The concentrations of the analytes in the resin were determined by HPLC as 3.1%, 4.6%, 1.9%, and 9.9%, respectively.

## Introduction

It is commonly accepted that a cholinergic deficit correlates with the severity of Alzheimer’s disease [1]. Thus, restoring cholinergic function is considered as a rational approach to slow down the progress of Alzheimer’s disease. One therapeutic option is the use of acetylcholinesterase (AChE) inhibitors which block this key enzyme in the breakdown of acetylcholine [2].

During the last two decades, the use of herbal medicinal drugs in dementia therapy has been studied based on experience from traditional medicine [3]. Using this knowledge, e.g. galanthamine from *Galanthus nivalis* L. (snowdrop) has been identified as a reversible, competitive AChE inhibitor and allosteric potentiating ligand of nicotinic acetylcholine re-ceptors and is today one therapeutic option in the treatment of Alzheimer’s disease [4, 5].

*Dorema ammoniacum* D. Don (Apiaceae) is a perennial plant in Iran, Afghanistan, and northern India. The gum-resin, commonly known as gum ammoniacum, which is secreted from damaged stems and roots, has been traditionally used as an expectorant, stimulant, and antispasmodic drug in the Unani system of medicine [6]. It is also used as an anthelmintic and for gastrointestinal disorders in Iranian traditional medicine (ITM) [7]. Some biological activities such as antibacterial and vasodilatory effects have been reported for this resin [6, 8]. Recently, a low cytotoxic activity was shown for the essential oil from fruits of *D. ammoniacum*[9]. Additionally, it is listed in the British Herbal Pharmacopoeia as an antispasmodic and expectorant, and it is used occasionally for chronic bronchitis and persistent coughs [10, 11].

A previous screening study on selected medicinal plants and plant products used in ITM showed AChE inhibitory activity for a dichloromethane (DCM) extract of gum ammoniacum [12]. Although the bioactivity of the extract was not very high, distinct bands from compounds with AchE inhibitory activity were detected by TLC-bioautography. Thus, the aim of this study was the isolation and characterization of these active compounds from the gum-resin.

## Results and Discussion

The gum-resin of *Dorema ammoniacum* has been used in ITM for centuries for different indications. There are several herbal mixtures for which a use in memory enhancement or treatment of memory loss is described in ITM. As most of these herbal preparations contain one or more gum-resins, gum ammoniacum had been included in a screening study for AChE inhibition [12]. Based on the detection of several active bands in TLC bioautography in the mentioned study, the DCM extract of gum ammoniacum was fractionated and four active compounds from this gum-resin were isolated and their structures characterized.

### Isolation of Active Compounds

By VLC a very fast enrichment of the active fractions from the extract was achieved. Subsequent column chromatography resulted in thirteen fractions of which C10 and C13 contained two and one active compounds, respectively, as monitored in TLC bio-autography. For the final step in the isolation of compounds **1** and **2,** HPCCC was performed on C10 using the solvent system hexane-ethylacetate-methanol-water (5+1+5+1) in normal phase elution, which yielded two oily components. Compound **3** was isolated from C13 by SPE on a C18 stationary phase under elution with MeOH–H_2_O (see “Experimental”). Compound **4** was purified as a single substance from fraction A10 from the first VLC via an HPCCC separation by normal phase elution and the use of the solvent system hexane-ethylacetate-methanol-water (5+2+5+2). Compound **4** was obtained in pure form after a final SPE step.

### Structure Elucidation of Active Compounds

ESI-MS measurements yielded a molecular weight of 426.0 Da for both **1** and **2**, and HR-ESI-MS showed an [M+H]^+^ ion at *m/z* 427.2125 for compound **1**, which is in agreement with the molecular formula C_25_H_30_O_6_ (calc’d for C_25_H_30_O_6_, [M+H]^+^, 427.2115, Δ = 2.4 ppm). In the positive ion mode ESI-MS^3^ spectra, major fragment ions were obtained at m/z 326.7 and 258.7 for **1** and **2**, respectively.

**Fig. 1:**
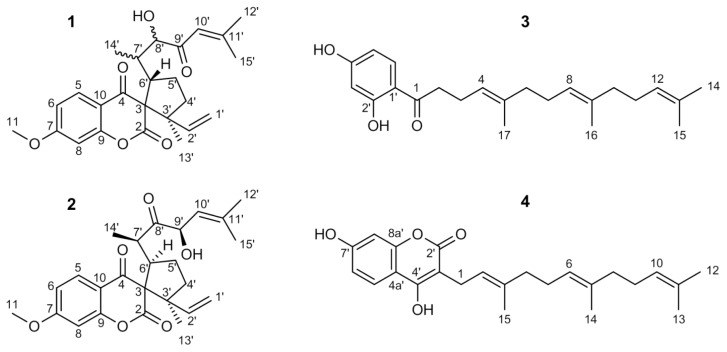
Structures of compounds **1–4**

The detailed ^1^H and ^13^C NMR analyses of **2,** together with numerous connectivities derived from 2D NMR spectra like COSY, TOCSY-NOESY, HSQC, as well as HMBC, resulted in the structure of a spiro-sesquiterpenoidic chromane-2,4-dione derivative, namely (2′*S*,5′*R*)-2′-ethenyl-5′-[(2*R*,4*R*)-4-hydroxy-6-methyl-3-oxohept-5-en-2-yl]-7-methoxy-2′-methyl-4*H*-spiro[chromene-3,1′-cyclopentane]-2,4-dione which is known as doremone A (Figure 1). All ^1^H as well as ^13^C chemical shifts match exactly with the published data [13]. The relative stereochemistry at carbon 3′ and 6′ could be confirmed by a NOESY crosspeak from H-6′ to the methyl group 13′. The configuration of the stereocentres 7′ and 9′ were proven by comparing the NMR data with those of the acetylated doremone A given by Appendino *et al*. [14] who in addition published an X-ray structure of the 3′ racemic mixture of acetyldoremone A [14].

The NMR spectra of **1** revealed a lot of similarities to those of **2**. All ^1^H and ^13^C NMR signals for the 7-methoxychromane-2,4-dione part showed almost the same chemical shifts including the spirane carbon with its very characteristic downfield shift to about 72 ppm. All other NMR signals varied from **2**, most predominantly in the sidechain. The ^13^C ppm value of the ketone shifts from 215 ppm in doremon A for almost 14 ppm to 201.4 ppm in **1** was indicative of a conjugation with the double bond. The corresponding olefinic proton 10′ in **1** was deshielded 1 ppm in the ^1^H NMR as compared to **2,** showing a septett multiplicity due to the allylic coupling with only two of the methyl groups. The vicinal coupling as in **2** was lost. The methyl protons 14′ in **1** gave an HMBC correlation to C-8′, which is a CH signal bearing an alcohol functionality due to a carbon shift of 79.8 ppm and a ^1^H shift of the attached proton of 3.90 ppm. As a result, these differences in the NMR spectra of the two compounds prove that the ketone at 8′ and the alcohol at 9′ in doremon A switched their positions in **1**, so that the carbonyl 9′ became conjugated with the double bond and the alcohol moved to 8′. The stereochemistry of these two centers remains undefined. The small shift variations in the cyclopentane part are not only due to the altered sidechain; a NOESY crosspeak between H-6′ and the olefinic H-2′ reveals a change in the relative configuration of the stereocentres 3′ and 6′ compared to doremone A. Based on these data, **1** was unambiguously identified as (2′*S*,5′*S*)-2′-ethenyl-5′-(3hydroxy-6-methyl-4-oxohept-5-en-2-yl)-7-methoxy-2′-methyl-4*H*-spiro[chromene-3,1′-cyclopentane]-2,4-dione (Figure 1).

The molecular weights of 356.1 and 382.1 Da were determined by ESI-MS for **3** and **4**, respectively, which are in agreement with the molecular formulae C_23_H_32_O_3_ and C_24_H_30_O_4_. The NMR data approved **3** as (4E,8E)-1-(2,4-dihydroxyphenyl)-5,9,13-trimethyltetradeca-4,8,12-trien-1-one (dshamirone) and **4** as 4,7-dihydroxy-3-[(2*E*,6*E*)-3,7,11-trimethyldo-deca-2,6,10-trien-1-yl]-2*H*-chromen-2-one (ammoresinol) (Figure 1). The NMR data of **3** were in agreement with an earlier publication [15]. However, this is the first report of this compound in the genus Dorema. Ammoresinol is a known compound from gum ammonia-cum, but there were no complete NMR data available for this substance until now [16, 17]. Thus, the detailed assignments for all ^1^H and ^13^C shifts for this compound are given in this study for the first time.

### AChEIinhibition

The AChE inhibitory activities of the DCM extract and the isolated compounds were determined for the first time in a microplate assay. The tested concentrations of the samples ranged from 1.56 to 200 μg/mL. Based on the resulting curves, the IC_50_ values for AChE inhibition were calculated using the respective curves’ equations (Table 1). Compounds **1**, **2,** and **4** showed only low activities, while compound **3** was about 10 times more active.

### Quantification of the Active Compounds

**Fig. 2:**
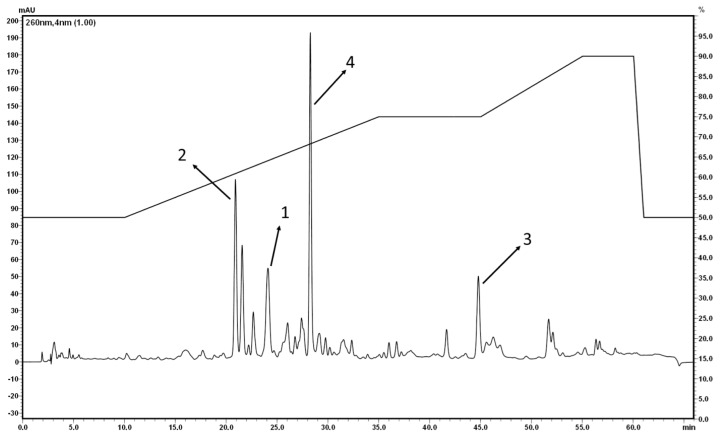
HPLC of the DCM extract; conditions see “Experimental”

HPLC analyses were performed for the quantification of the isolated components in the tested extract and gum ammoniacum. Several HPLC conditions under variation of the mobile phase, flow rate, and elution gradient were tested to optimize the separation of compounds **1–4** in the DCM extract (Figure 2).

The concentrations of the active compounds were determined by external standardization. The peak areas of all compounds were linearly dependent of the concentration over the selected range. The concentrations of the active compounds in the DCM extract and gum ammoniacum as well as the correlation coefficients of the linear regressions in external standardization are summarized in Table 2.

## Conclusion

A new compound and three known compounds were isolated from gum ammoniacum and their AChE inhibitory activities were determined for the first time. From the correlation of their IC_50_ values with their concentrations in gum ammoniacum, it can be concluded that compounds **1** to **4** are contributing to the AChE inhibition of this drug. Due to these results, the use of this drug in ITM in mixtures improving cognitive functions seems plausible.

## Experimental

### Chemicals

AChE from electric eel, 1-naphthyl acetate, 5,5′-dithiobis-(2-nitrobenzoic acid) (DTNB), Tris-HCl, bovine serum albumin (BSA), and physostigmine were purchased from Sigma (St. Louis, USA). Acetylthiocholine iodide (ATCI) and chelidonine were obtained from Fluka (Buchs, Switzerland). Fast Blue B salt (FBS) and silica gel 60 were from Merck (Darmstadt, Germany). The solvents MeOH, EtOAc, CH_2_Cl_2,_ and CHCl_3_ were purchased from VWR (Vienna, Austria). Two different buffer systems were used (buffer A: 50 mM Tris-HCl, pH 7.9 containing 0.1% BSA; buffer B: 50 mM Tris-HCl, pH 7.9 containing 0.1 M NaCl and 0.02 M MgCl_2_·6H_2_O).

### General

A Genios microplate reader (Tecan, Salzburg, Austria) was used to measure the absorbance. The extraction was performed by sonification in a Branson 3150 ultrasonic bath (Dumbury, USA). The silica gel 60 F_254_ TLC plates were obtained from Merck (Darmstadt, Germany) and 96-well microplates PS F-bottom from Greiner Bio-One (Frickenhausen, Germany). For solid-phase extraction (SPE), Mega Bond Elut-C18 cartridges from Varian (Santa Clara, USA) were used. In the LC-MS analyses the separation was performed on an Acclaim 120 C18 column, 2.1 × 150 mm, 3 μm (Dionex, Germering, Germany) using an UltiMate 3000 RSLC-series system (Dionex, Germering, Germany) coupled to a 3D quadrupole ion trap mass spectrometer via an orthogonal ESI source (HCT, Bruker Daltonics, Bremen, Germany). The HR-ESI-MS spectra were recorded on an ESI-Qq-TOF mass spectrometer (micrOTOF-Q II; Bruker, Daltonics) in positive ion mode by direct infusion. The UV-VIS spectra and CD spectra were measured on the Spectropolarimeter Jasco J-810 (QS 1 mm; Cremella, Italy). The infrared spectra were measured on the Perkin Elmer FT-IR 2000 instrument (Waltham, USA) in attenuated total reflection mode using a Golden Gate ATR unit. All NMR spectra were recorded on the Bruker Avance DRX 600 spectrometer (Bruker BioSpin, Rheinstetten, Germany). The Spectrum HPCCC instrument (Dynamic Extractions, Berkshire, UK) was used for high-performance counter current-chromatography (HPCCC). HPLC was performed on the Shimadzu instrument with LC-20AD pump, SPD M20A diode array detector and SIL 20AC HT autosampler (Kyoto, Japan). The EZ-2plus evaporator from Genevac (New York, USA) was used to evaporate the solvents.

### Drug Material

The dry gum-resin of *Dorema ammoniacum* D. Don was purchased from an herbal shop in Tehran, Iran, and identified by Dr. Gholamreza Amin at the herbarium of the Faculty of Pharmacy, Tehran University of Medical Sciences, Tehran, Iran (voucher number PMP-804).

### Extraction

Twenty grams of the resin were ground and extracted twice by sonification with 200 mL DCM at 40°C for 1 hour. The extracts were combined and concentrated under reduced pressure at 40° C to yield 12.98 g of the DCM extract.

### TLC Bioautography Assay

The DCM extract of gum ammoniacum was examined by TLC on silica plates using the mobile phase chloroform-ethylacetate-methanol (90+7+3). Anisaldehyde-sulfuric acid was used as the detection reagent to determine the chemical composition of the extract [18]. In parallel, a TLC bioautography assay was performed for the AChE inhibitory activity according to a published method [19, 20]. The mobile phase was completely removed under airstream before detection. Chelidonine served as a positive control in TLC showing R*_F_* 0.42 in this TLC system.

### Isolation of Active Compounds

Fractionation of 12.0 g DCM extract of gum ammoniacum was performed by vacuum liquid chromatography (VLC) with silica gel 60 and chloroform as the stationary and mobile phase, respectively. Fractions (500 mL/10 min) with similar chemical composition according to TLC were combined. Twelve collective fractions (A1–A12) were examined by TLC bioautography for their AChE inhibitory activity. The active compounds were isolated from these fractions.

Compounds **1** and **2**: Further purification of the active fraction A4 (4.25 g) using VLC on a silica gel column under gradient elution with 100–50% petroleum ether in chloroform yielded five fractions (B1–B5). Then 2.85 g of the active fraction B5 was eluted with 50% petroleum ether in chloroform. Two grams of B5 were loaded on a silica gel column and eluted with chloroform (10 mL/30 min). From the resulting 13 collective fractions (C1–C13), the two active ones were fractions C10 (468 mg; combined from subfractions 175 to 179) and C13 (94 mg; combined from subfractions 185 to 189). An HPCCC under normal phase elution applying the solvent system hexane–ethylacetate–methanol–water (5+1+5+1) was carried out for 360 mg of C10 to obtain the two oily active compounds **1** (6.7 mg; eluting from 107 to 118 min) and **2** (119 mg; eluting from 147 to 175 min). The rotation speed of HPCCC was 1620 rpm and the fraction size 6 mL/min.

Compound **3** was isolated by solid phase extraction of 90 mg of fraction C13 (on a Mega Bond Elut C-18 cartridge (volume 12 mL). The mobile phase consisted of methanol-water and a flow rate of 2.5 mL/min was applied. The methanol concentration was increased stepwise from 40% to 90% in 10% steps. For each concentration, five bed volumes were used for elution. In the elution step with 80% MeOH, 9.6 mg of compound 3 were obtained.

Compound **4**: 1.7 g of fraction A10 was further fractionated by HPCCC using normal phase elution to obtain 9 collective fractions (M1–M9). The solvent mixture consisted of hexane–ethylacetate–methanol–water (5+2+5+2). A solid phase extraction was performed for 100 mg of fraction M9 (eluted in HPCCC from 45 to 63 min) on a Mega Bond Elut C-18 cartridge under elution with 40–90% methanol at a flow rate of 2.5 mL/min as described above. Finally, 4.1 mg of compound 4 were obtained under elution with 80% MeOH.

### Structure Characterization of Active Compounds

The separation in the LC-MS analysis was carried out at 25°C and a flow rate of 0.5 mL/min. A solution of 0.1% aqueous formic acid and acetonitrile were used as mobile phase A and B, respectively. Compounds **1** and **2** were eluted by gradient elution: 40% B (0 min), 40% B (2 min), and 70% B (32 min), while the following gradient program was used for compounds **3** and **4**: 75% B to 82% B in 14 min. The eluent flow was split roughly 1:8 before the ESI ion source, which was operated as follows: capillary voltage: −3.7/+3.5 kV, nebulizer: 26 psi (N_2_), dry gas flow: 9 L/min (N_2_), and dry temperature: 340°C. Collision-induced dissociation (CID) spectra were obtained in automated data-dependent acquisition (DDA) mode with helium as the collision gas, an isolation window of 4 Th, and a fragmentation amplitude of 1.0 V.

Compound **1:** (+)ESIMS *m/z* 427.0 [M+H]^+^; ESIMS^2^ (427.0 →) *m/z* 408.8 (100), 326.7 (14); ESIMS^3^ (427.0 → 408.8 →) *m/z* 326.7 (100), 298.7 (12), 284.7 (51), 204.6 (19), 150.7 (13); (−)ESIMS *m/z* 425.0 [M-H]^−^; ESIMS^2^ (425.0 →) *m/z* 380.8 (11), 362.8 (16), 204.6 (100); HR-ESI-MS *m/z* 427.2125 [M+H]^+^ (calcd for C_25_H_30_O_6_, [M+H]^+^, 427.2115, Δ = 2.4 ppm); CD (CHCl_3_) Δ*ɛ*_253_ +1.92, Δ*ɛ*_275_ −4.24, Δ*ɛ*_297_ +0.24, Δ*ɛ*_320_ −2.59; UV λ_max_ (CHCl_3_) 240.1, 275.0, 308.2 (sh) nm; IR ν_max_ 3184, 2359, 1626, 1550, 1296, 1037, 663 cm^−1^.

Compound **2**: (+)ESIMS *m/z* 427.0 [M+H]^+^; ESIMS^2^ (427.0 →) *m/z* 408.8 (100), 258.7 (11); ESIMS^3^ (427.0 → 408.8 →) *m/z* 390.8 (57), 352.7 (38), 334.7 (25), 326.7 (63), 310.6 (21), 298.7 (14), 284.7 (27), 270.6 (17), 258.6 (100), 240.6 (11), 230.7 (11), 218.6 (18), 216.7 (15), 204.6 (63), 192.7 (22), 174.8 (11), 150.7 (18); (−)ESIMS *m/z* 425.0 [M-H]^−^; ESIMS^2^ (425.0 →) *m/z* 380.8 (28), 362.8 (15), 256.7 (100), 166.6 (20), 150.7 (24).

Compound **3**: (+)ESIMS *m/z* 357.1 [M+H]^+^; ESIMS^2^ (357.1 →) *m/z* 339.1 (100), 229.1 (16), 203.1 (22), 165.0 (11), 149.0 (20), 147.1 (10), 137.0 (11), 123.1 (29); (−)ESIMS *m/z* 355.1 [M-H]^−^; ESIMS^2^ (355.1 →) *m/z* 217.9 (10), 149.9 (100), 135.9 (12), 122.0 (26); ESIMS^3^ (355.1 → 149.9 →) *m/z* 122.0 (100).

Compound **4**: (+)ESIMS *m/z* 383.1 [M+H]^+^; ESIMS^2^ (383.1 →) *m/z* 190.9 (100); ESIMS^3^ (383.1 → 190.9 →) *m/z* 162.9 (17), 123.0 (100); (−)ESIMS *m/z* 381.1 [M-H]^−^; ESIMS^2^ (381.1 →) *m/z* 254.9 (17), 243.9 (34), 242.9 (33), 228.9 (16), 202.9 (11), 189.9 (51), 188.9 (100), 175.9 (62).

All NMR spectra were recorded on a Bruker Avance DRX 600 NMR spectrometer using a 5 mm switchable quadruple probe (QNP, ^1^H, ^13^C, ^19^F, ^31^P) with z axis gradients and automatic tuning and a matching accessory. The resonance frequency for ^1^H NMR was 600.13 MHz, for ^13^C NMR 150.92 MHz. All measurements were performed on a solution in CDCl_3_ at 298K. Standard 1D and gradient-enhanced (ge) 2D experiments, like double-quantum filtered (DQF) COSY, TOCSY, NOESY, HSQC, and HMBC, were used as supplied by the manufacturer. Chemical shifts are referenced internally to the residual, non-deuterated solvent signal for ^1^H (δ = 7.26 ppm) or to the carbon signal of the solvent for ^13^C (δ = 77.0 ppm). The ^1^H and ^13^C NMR data are summarized in Tables 3 and 4.

### Microplate Assay

A quantitative colorimetric assay based on Ellman’s method was used to measure the AChE inhibitory activities of the substances [20, 21]. Briefly, in a 96-well plate, 25 μL of 15 mM ATCI, 125 μL of 3 mM DTNB in buffer B, 50 μL of buffer A, and 25 μL of the extract or the isolated substances (from 15.6 μg/mL to 2.0 mg/mL in 10% DMSO) were thoroughly mixed and the absorbance was read at 405 nm five times every 15 s. Finally, 25 μL of AChE (0.22 U/mL in buffer A) were added and the plate was incubated at 25°C for 10 minutes. Then the absorbance was measured again eight times every 15 s. A 10% DMSO solution was used as the negative control. To compensate for any increase in absorbance due to the color of the extracts or spontaneous hydrolysis of the substrate, the absorbance before the addition of the enzyme was subtracted from the absorbance after adding the enzyme. The assay was repeated three times for every concentration. Physostigmine at different concentrations (0.12–15 μg/mL) served as the positive control. The IC_50_ value of physostigmine was determined as 0.80 ± 0.04 μg/mL (2.91 μM).

### HPLC

The DCM extract of gum ammoniacum and the isolated compounds were analyzed by HPLC using gradient elution on the Hypersil BDS-C18 column (250×4 mm id) at 35°C. The mobile phase consisted of acetonitrile (A) and 0.05% formic acid in water (B) at a flow rate of 0.75 mL/min. After an isocratic step at 50% A for 10 min, the concentration of solvent A increased up to 75% in 25 min. The mobile phase was kept at this concentration for 10 min, increased to 90% A in 10 min, and was kept again at 90% for a further 5 min. Elution was monitored at 260 nm.

## Figures and Tables

**Tab. 1:** Inhibition of AchE by the DCM extract, compounds **1–4** and the positive control physostigmine (n=3)

Substance	IC_50_ (μg/mL)	IC_50_ (μM)
**1**	77.14 ± 3.75	181.08
**2**	100.82 ± 5.14	236.66
**3**	8.36 ± 0.41	23.50
**4**	76.84 ± 3.86	200.90
DCM extract	668.00 ± 17.80	–
Physostigmine	0.80 ± 0.04	2.91

**Tab. 2:** Content of compounds **1** to **4** in the DCM extract and in gum ammoniacum and correlation coefficients for external standardization

Substance	% in DCM extract	% in gum ammonicum	Correlation coefficients
1	5.8	3.1	R^2^ = 0.9869
2	8.4	4.6	R^2^ = 0.9837
3	3.1	1.9	R^2^ = 0.9959
4	15.9	9.9	R^2^ = 0.9854

**Tab. 3:** ^1^H (600 MHz) and ^13^C NMR (150 MHz) of compounds **1** and **2** (CDCl_3_)

	Compound 1		Compound 2	

Atom^a^	δ_C_ (dept)	δ_H_	δ_C_ (dept)	δ_H_
2	169.8 (s)	–	168.4 (s)	–
3	70.8 (s)	–	72.1 (s)	–
4	191.4 (s)	–	189.2 (s)	–
5	127.9 (d)	7.85 (1H, d, *J*= 8.8 Hz)	128.6 (d)	7.80 (1H, d, *J*= 8.8 Hz)
6	111.7 (d)	6.76 (1H, dd, *J*= 8.8, 2.4 Hz)	112.2 (d)	6.75 (1H, dd, *J*= 8.8, 2.4 Hz)
7	165.5 (s)	–	166.2 (s)	–
8	100.7 (d)	6.58 (1H, d, *J*= 2.4 Hz)	100.8 (d)	6.57 (1H, d, *J*= 2.4 Hz)
9	156.5 (s)	–	156.3 (s)	–
10	115.4 (s)	–	113.8 (s)	–
11	55.8 (q)	3.86 (3H, s)	55.6 (q)	3.88 (3H, s)
1′	112.9 (t)	5.07 (1H, dd, *J*= 17.3, 0.8 Hz) 5.13 (1H, dd, *J*= 10.8, 0.8 Hz)	115.9 (t)	4.90 (1H, dd, *J*= 10.8, 0.7 Hz) 4.89 (1H, dd, *J*= 17.3, 0.7 Hz)
2′	142.7 (d)	5.96 (1H, dd, *J*= 17.3, 10.8 Hz)	140.2 (d)	5.60 (1H, dd, *J*= 17.3, 10.8 Hz)
3′	56.1 (s)	–	56.2 (s)	–
4′	34.2 (t)	1.86 (2H, m)	35.9 (t)	2.36 (1H, m) 1.54 (1H, ddd, *J*= 12.9, 5.5, 2.7 Hz)
5′	29.6 (t)	2.21 (1H, m) 1.93 (1H, m)	28.4 (t)	2.25 (1H, m) 1.87 (1H, m)
6′	49.4 (d)	3.03 (1H, ddd, *J*= 9.0, 10.5, 10.5 Hz)	46.1 (d)	3.44 (1H, ddd, *J*= 8.0, 10.4, 10.4 Hz)
7′	39.7 (d)	1.93 (1H, m)	44.7 (d)	3.03 (1H, qd, *J*= 7.0, 10.4 Hz)
8′	79.8 (d)	3.90 (1H, dd, *J*= 7.8, 9.6 Hz) 2.77 (1H: OH, d, *J*= 7.8 Hz)	215.0 (s)	–
9′	201.4 (s)	–	73.9 (d)	4.69 (1H, br d, *J*= 9.8 Hz) 3.41 (1H: OH, br)
10′	122.7 (d)	6.01 (1H, sep, *J*= 1.1 Hz)	120.0 (d)	4.99 (1H, sep d, *J*= 1.4, 9.8 Hz)
11′	158.7 (s)	–	140.3 (s)	–
12′	28.1 (q)	1.90 (3H, d, *J*= 1.1 Hz)	26.0 (q)	1.80 (3H, d, *J*= 1.4 Hz)
13′	22.2 (q)	0.90 (3H, s)	23.2 (q)	0.98 (3H, d, *J*= 0.6 Hz)
14′	16.1 (q)	0.92 (3H, d, *J*= 6.9 Hz)	15.7 (q)	1.21 (3H, d, *J*= 7.0 Hz)
15′	21.4 (q)	2.10 (3H, d, *J*= 1.1 Hz)	18.5 (q)	2.10 (3H, d, *J*= 1.4 Hz)

a for numbering see figure 1.

**Table 4:** ^1^H (600 MHz) and ^13^C NMR (150 MHz) of compounds **3** and **4** (CDCl_3_)

Compound 3			Compound 4		

Atom^a^	δ_C_ (dept)	δ_H_	Atom^a^	δ_C_ (dept)	δ_H_
1	204.7 (s)	–	1	23.7 (t)	3.41 (2H, dq, *J=* 7.6, 0.6 Hz)
2	38.1 (t)	2.92 (2H, m)	2	120.2 (d)	5.45 (1H, tq, *J=* 7.6, 1,4 Hz)
3	23.2 (d)	2.42 (1H, m)	3	142.8 (s)	–
4	122.3 (d)	5.17 (1H, tqt, *J=* 7.2, 1.4, 1.2 Hz)	4	39.7 (t)	2.17 (2H, m)
5	136.8 (s)	–	5	26.0 (s)	2.17 (2H, m)
6	39.6 (t)	1,99 (2H, m)	6	123.0 (d)	5.01 (1H, m)
7	26.5 (t)	2,06 (2H, m)	7	136.4 (s)	–
8	124.0 (d)	5,09 (1H, m)	8	39.7 (t)	2,01 (2H, m)
9	135.1 (s)	–	9	26.6 (t)	2.07 (2H, m)
10	39.7 (t)	1.97 (2H, m)	10	124.2 (d)	5.08 (1H, m)
11	26.7 (t)	2.06 (2H, m)	11	131.4 (s)	–
12	124.4 (d)	5.09 (1H, m)	12	25.7 (q)	1.67 (3H, m)
13	131.3 (s)	–	13	17.7 (q)	1.58 (3H, m)
14	25.7 (q)	1.68 (3H, m)	14	16.4 (q)	1.62 (3H, m)
15	17.7 (q)	1.60 (3H, m)	15	16.4 (q)	1.84 (3H, m)
16	16.0 (q)	1.59 (3H, m)	2′	164.9 (s)	–
17	16.0 (q)	1.66 (3H, m)	3′	99.7 (s)	–
1′	113.9 (s)	–	4′	162.4 (s)	–
2′	165.2 (s)	−12.83 (1H,breit)	4a′	108.9 (s)	–
			5′	124.2 (d)	7.63 (1H, d, *J=* 8.7 Hz)
3′	103.5 (d)	6.38 (1H, m)	6′	113.2 (d)	6.82 (1H, dd*, J=* 8.7, 2.4 Hz)
4′	162.5 (s)	–	7′	160.0 (s)	–
5′	107.6 (d)	6.37 (1H, m)	8′	102.6 (d)	6.97 (1H,d*, J=* 2,4 Hz)
6′	132.4 (d)	7.65 (1H, d, *J=* 9.3 Hz)	8a′	153.9 (s)	–

a for numbering see figure 1.
